# Poly[[(μ-benzene-1,4-dicarboxyl­ato)bis­[μ-4-(1*H*-1,3,7,8-tetra­aza­cyclo­penta­[*l*]phenanthren-2-yl)benzoato]dizinc] tetra­hydrate]

**DOI:** 10.1107/S1600536811040062

**Published:** 2011-10-05

**Authors:** Chun-Jie Wang, Qian-Qian Wang, Xiang-Jun Xu, Chun-Bo Liu, Guang-Bo Che

**Affiliations:** a School of Chemistry and Chemical Engineering, Jiangsu University, Zhenjiang 212013, People’s Republic of China

## Abstract

In the title complex, [Zn_2_(C_8_H_4_O_4_)(C_20_H_11_N_4_O_2_)_2_]·4H_2_O, the Zn^II^ atom is six-coordinated by two carboxyl­ate O atoms from one bidentate benzene-1,4-dicarboxyl­ate (1,4-BDC) ligand, two carboxyl­ate O atoms from two different monodentate 4-(1*H*-1,3,7,8-tetra­aza­cyclo­penta­[*l*]phenanthren-2-yl)benzoate (HNCP) ligands and two HNCP N atoms. The Zn^II^ atoms are bridged by the centrosymmetric 1,4-BDC ligands, forming an extended single-chain structure. Neighbouring single chains are connected by the HNCP ligands from two opposite directions, resulting in a sheet. In addition, there are N—H⋯O hydrogen-bonding inter­actions between adjacent layers. As a result, the polymeric sheets are further extended into a three-dimensional supra­molecular structure.

## Related literature

For the preparation of the 4-(1*H*-1,3,7,8-tetra­aza­cyclo­penta­[*l*]phenanthren-2-yl)benzoate (HNCP) ligand, see: Yongqin *et al.* (2007 ▶). For coordination polymers with a variety of supra­molecular structures, see: Eddaoudi *et al.* (2001 ▶); Chen & Liu (2002 ▶). For HNCP-based complexes, see Yongqin *et al.* (2007 ▶); Hsu *et al.* (2005 ▶). 
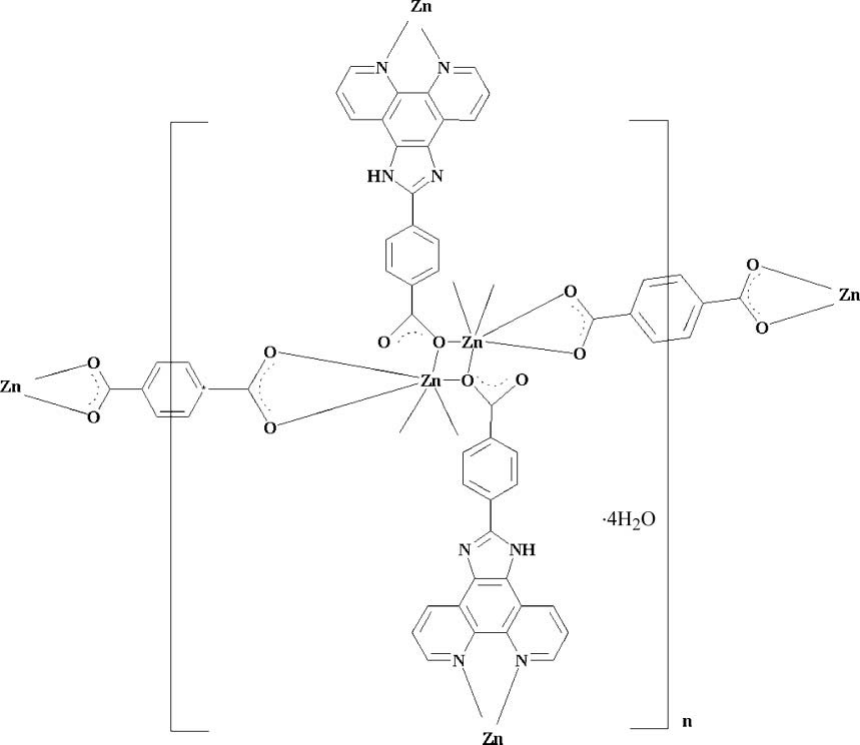


## Experimental

### 

#### Crystal data

[Zn_2_(C_8_H_4_O_4_)(C_20_H_11_N_4_O_2_)_2_]·4H_2_O
*M* *_r_* = 1037.54
Triclinic, 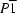

*a* = 9.7477 (16) Å
*b* = 10.2610 (18) Å
*c* = 11.0480 (19) Å
α = 88.849 (3)°
β = 72.115 (4)°
γ = 83.121 (3)°
*V* = 1043.9 (3) Å^3^
*Z* = 1
Mo *K*α radiation
μ = 1.23 mm^−1^
*T* = 293 K
0.2 × 0.2 × 0.2 mm

#### Data collection

Bruker SMART CCD area-detector diffractometer
Absorption correction: multi-scan (*SADABS*; Bruker, 2002 ▶) *T*_min_ = 0.782, *T*_max_ = 0.782
5406 measured reflections
3783 independent reflections
2797 reflections with *I* > 2σ(*I*)
*R*_int_ = 0.029

#### Refinement

*R*[*F* ^2^ > 2σ(*F* ^2^)] = 0.056
*wR*(*F* ^2^) = 0.139
*S* = 1.02
3783 reflections
316 parameters
H-atom parameters constrained
Δρ_max_ = 0.94 e Å^−3^
Δρ_min_ = −0.66 e Å^−3^

### 

Data collection: *SMART* (Bruker, 2002 ▶); cell refinement: *SAINT* (Bruker, 2002 ▶); data reduction: *SAINT*; program(s) used to solve structure: *SHELXS97* (Sheldrick, 2008 ▶); program(s) used to refine structure: *SHELXL97* (Sheldrick, 2008 ▶); molecular graphics: *SHELXTL* (Sheldrick, 2008 ▶); software used to prepare material for publication: *SHELXTL*.

## Supplementary Material

Crystal structure: contains datablock(s) global, I. DOI: 10.1107/S1600536811040062/bg2420sup1.cif

Structure factors: contains datablock(s) I. DOI: 10.1107/S1600536811040062/bg2420Isup2.hkl

Additional supplementary materials:  crystallographic information; 3D view; checkCIF report

## Figures and Tables

**Table 1:** Hydrogen-bond geometry (Å, °)

*D*—H⋯*A*	*D*—H	H⋯*A*	*D*⋯*A*	*D*—H⋯*A*
N3—H4⋯O3^i^	0.86	2.10	2.781 (5)	136

Symmetry code: (i) 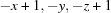.

**Fig. 1.**
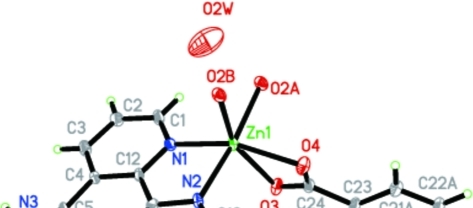
The asymmetric unit of (I), together with further atoms to complete the Zn1 coordination and the 1,4-BDC ligand. Displacement ellipsoids are drawn at the 30% probability level. (arbitrary spheres for the H atoms). Atoms O2A, O2B and C21A/C22A/C23A/C24A/O3A/O4A are generated by the symmetry code (x, -y + 1, -z + 1), (-x + 1, -y, -z) and (-x, -y + 1, -z + 2), respectively.

**Fig. 2.**
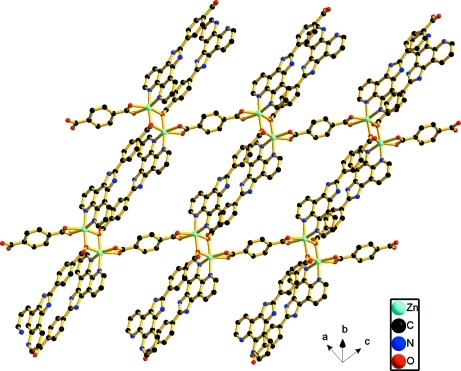
View of the two-dimensional sheet formed by HNCP linked linear chain. H atoms have been omitted.

**Fig. 3.**
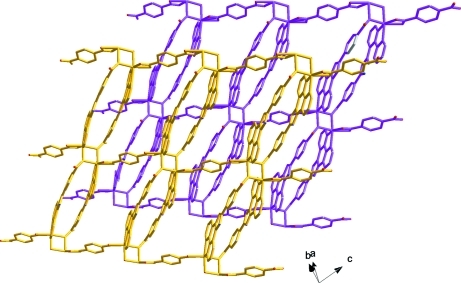
A view of the three-dimensional structure of 1 linked by hydrogen bonds between the adjacent sheets. H atoms have been omitted.

## supplementary crystallographic information

### Comment

Coordination polymers with a variety of supramolecular structures have been studied extensively because of their novel topologies and potential applications as functional materials (Eddaoudi *et al.*, 2001). 1,10-Phenanthroline (phen), as a common organic ligand, has been widely used in the construction of metal-organic coordination polymers (Chen & Liu, 2002). The phen derivative 4-(1*H*-1, 3, 7, 8-Tetraaza-cyclopenta [*l*]phenanthren-2-yl)-benzoic acid (HNCP) with both phenanthroline ring and carboxylate groups, is a good building block for construction of coordination polymers. Moreover, the long-conjugated system and the carboxylate groups are inclined to form π-π stacking interactions and hydrogen bonding interactions, which are important factors in the formation of supramolecular architectures. However, to date, only a handful of supramolecular architectures based on HNCP molecules have been described (Yongqin *et al.*, 2007; Hsu *et al.*, 2005). We selected benzene-1,4-dicarboxylic acid (1,4-BDC) as a linker and HNCP as a secondary ligand, generating a new coordination polymer, [Zn_2_(1,4-BDC)_(NCP)_2_(H_2_O)_4] (I), which is reported here.

In compound (I), the Zn atom is coordinated by two N atoms from one HNCP ligand, two O atoms from one 1,4-BDC ligand, and two O atoms from two different HNCP ligands in a distorted octahedral coordination (Fig. 1). The single unique 1,4-BDC species is generated from the atoms of the asymmetric unit by inversion. Two Zn^II^ centers are bridged by the carboxylate groups of HNCP ligands to furnish a binuclear unit with a Zn^II^···Zn^II^ distance of 3.2224 (9) Å. Neighbouring binuclear units are bridged by 1,4-BDC ligands, forming a single-chain structure. The neighboring single chains constructed by the 1,4-BDC ligand and the Zn^II^ atoms are connected by the HNCP ligands from two opposite directions and result in a two-dimensional sheet (Fig. 2).

Even though the H atoms pertaining to water molecules could not be confidently found, and accordingly were not included in the model, the short O1···O2W^i^ 2.891 (9)Å; O1W···O2W^ii^ 2.886 (14)Å; O1W···N4: 2.899 (10)Å distances, (i): x, -1+y, -1+z; (ii): 1-x,-y,1-z might indicate the formation of hydrogen bonds between these atoms.

Besides, there are hydrogen bonding interactions between adjacent layers. The imidazole nitrogen atoms of HNCP ligands act as hydrogen bond donors, while the carboxylate oxygen atoms of 1,4-BDC ligands from the neighboring layer act as hydrogen bond acceptors (N3—H···O3) (Table 1). As a result, these two-dimensional polymeric sheets are further extended into three-dimensional supramolecular structures through these hydrogen-bonding interactions(Fig. 3).

### Experimental

HNCP was prepared according to the literature method (Yongqin *et al.*, 2007). Other reagents were commercially available and used without further purification. Zn(CH_3_COO)2 (0.2 mmol), 1,4-BDC (0.1 mmol), and HNCP (0.1 mmol) were mixed in 10 ml deionized water. And its pH value was controlled in the range of 7–8 with 1 mol/*L* NaOH solution. Then, the resulting precursor was placed in 25 ml Teflon-lined stainless steel reactor, and heated at 433 K for 3 d. Cooling slowly to room temperature, the yellow block crystals of the title complex suitable for X-ray diffraction analysis were obtained.

### Refinement

All C- and N- attached H atoms were positioned geometrically (N—H = 0.86Å and C—H = 0.93 Å) and refined as riding, with *U*_iso_(H)= 1.2*U*_eq_(C). Hydrogen atoms corresponding to water molecules could not be confidently located and were not included in the model.

### Crystal data

**Table:**

[Zn_2_(C_8_H_4_O_4_)(C_20_H_11_N_4_O_2_)_2_]·4H_2_O	*Z* = 1
*M**_r_* = 1037.54	*F*(000) = 526
Triclinic, *P*1	*D*_x_ = 1.651 Mg m^−^^3^*D*_m_ = 1.651 Mg m^−^^3^*D*_m_ measured by not measured
Hall symbol: -P 1	Mo *K*α radiation, λ = 0.71073 Å
*a* = 9.7477 (16) Å	Cell parameters from 5729 reflections
*b* = 10.2610 (18) Å	θ = 2.9–25.3°
*c* = 11.0480 (19) Å	µ = 1.23 mm^−^^1^
α = 88.849 (3)°	*T* = 293 K
β = 72.115 (4)°	Block, yellow
γ = 83.121 (3)°	0.2 × 0.2 × 0.2 mm
*V* = 1043.9 (3) Å^3^	

### Data collection

**Table:**

Bruker SMART CCD area-detector diffractometer	3783 independent reflections
Radiation source: fine-focus sealed tube	2797 reflections with *I* > 2σ(*I*)
graphite	*R*_int_ = 0.029
ω scans	θ_max_ = 25.3°, θ_min_ = 1.9°
Absorption correction: multi-scan (*SADABS*; Bruker, 2002)	*h* = −11→10
*T*_min_ = 0.782, *T*_max_ = 0.782	*k* = −12→11
5406 measured reflections	*l* = −10→13

### Refinement

**Table:**

Refinement on *F*^2^	Primary atom site location: structure-invariant direct methods
Least-squares matrix: full	Secondary atom site location: difference Fourier map
*R*[*F*^2^ > 2σ(*F*^2^)] = 0.056	Hydrogen site location: inferred from neighbouring sites
*wR*(*F*^2^) = 0.139	H-atom parameters constrained
*S* = 1.02	*w* = 1/[σ^2^(*F*_o_^2^) + (0.0678*P*)^2^ + 0.7102*P*] where *P* = (*F*_o_^2^ + 2*F*_c_^2^)/3
3783 reflections	(Δ/σ)_max_ < 0.001
316 parameters	Δρ_max_ = 0.94 e Å^−^^3^
0 restraints	Δρ_min_ = −0.66 e Å^−^^3^

### Special details

**Table:**

Geometry. All esds (except the esd in the dihedral angle between two l.s. planes) are estimated using the full covariance matrix. The cell esds are taken into account individually in the estimation of esds in distances, angles and torsion angles; correlations between esds in cell parameters are only used when they are defined by crystal symmetry. An approximate (isotropic) treatment of cell esds is used for estimating esds involving l.s. planes.
Refinement. Refinement of F^2^ against ALL reflections. The weighted R-factor wR and goodness of fit S are based on F^2^, conventional R-factors R are based on F, with F set to zero for negative F^2^. The threshold expression of F^2^ > 2sigma(F^2^) is used only for calculating R-factors(gt) etc. and is not relevant to the choice of reflections for refinement. R-factors based on F^2^ are statistically about twice as large as those based on F, and R- factors based on ALL data will be even larger.

### Fractional atomic coordinates and isotropic or equivalent isotropic displacement parameters (Å^2^)

**Table:**

	*x*	*y*	*z*	*U*_iso_*/*U*_eq_	
Zn1	0.39808 (6)	0.38395 (5)	0.54113 (5)	0.0246 (2)	
C1	0.6671 (5)	0.1755 (5)	0.4727 (5)	0.0305 (12)	
H1	0.7021	0.2253	0.5235	0.037*	
C2	0.7546 (5)	0.0643 (5)	0.4098 (5)	0.0318 (12)	
H2	0.8454	0.0403	0.4200	0.038*	
C3	0.7059 (5)	−0.0093 (5)	0.3327 (5)	0.0304 (12)	
H3	0.7632	−0.0835	0.2900	0.036*	
C4	0.5686 (5)	0.0291 (4)	0.3194 (4)	0.0212 (10)	
C5	0.5041 (5)	−0.0321 (4)	0.2395 (4)	0.0237 (11)	
C6	0.3733 (5)	0.0143 (4)	0.2210 (5)	0.0252 (11)	
C7	0.2859 (5)	0.1260 (5)	0.2933 (5)	0.0253 (11)	
C8	0.1468 (5)	0.1777 (5)	0.2890 (5)	0.0333 (12)	
H8	0.1048	0.1402	0.2352	0.040*	
C9	0.0745 (6)	0.2835 (5)	0.3647 (5)	0.0380 (14)	
H9	−0.0181	0.3176	0.3641	0.046*	
C10	0.1403 (5)	0.3396 (5)	0.4423 (5)	0.0333 (13)	
H10	0.0903	0.4124	0.4924	0.040*	
C11	0.3431 (5)	0.1878 (4)	0.3759 (4)	0.0232 (10)	
C12	0.4856 (5)	0.1422 (4)	0.3874 (4)	0.0208 (10)	
C13	0.4616 (5)	−0.1529 (4)	0.0957 (5)	0.0243 (11)	
C14	0.4894 (5)	−0.2566 (4)	−0.0019 (5)	0.0255 (11)	
C15	0.6139 (5)	−0.3454 (5)	−0.0274 (5)	0.0299 (12)	
H15	0.6767	−0.3415	0.0207	0.036*	
C16	0.6468 (5)	−0.4398 (5)	−0.1230 (5)	0.0298 (12)	
H16	0.7298	−0.4998	−0.1371	0.036*	
C17	0.5563 (5)	−0.4452 (4)	−0.1980 (4)	0.0246 (11)	
C18	0.4302 (5)	−0.3563 (4)	−0.1718 (4)	0.0251 (11)	
H18	0.3680	−0.3587	−0.2206	0.030*	
C19	0.3971 (5)	−0.2648 (5)	−0.0741 (5)	0.0271 (11)	
H19	0.3114	−0.2079	−0.0566	0.033*	
C20	0.5974 (6)	−0.5445 (5)	−0.3046 (5)	0.0296 (12)	
C21	−0.0493 (5)	0.4022 (5)	1.0852 (5)	0.0307 (12)	
H23	−0.0820	0.3366	1.1424	0.037*	
C22	0.0434 (5)	0.3690 (5)	0.9641 (5)	0.0315 (12)	
H22	0.0721	0.2812	0.9401	0.038*	
C23	0.0935 (5)	0.4665 (5)	0.8785 (5)	0.0291 (12)	
C24	0.1997 (5)	0.4312 (5)	0.7506 (5)	0.0261 (11)	
N1	0.5364 (4)	0.2129 (4)	0.4629 (4)	0.0230 (9)	
N2	0.2706 (4)	0.2945 (4)	0.4484 (4)	0.0248 (9)	
N3	0.5581 (4)	−0.1389 (4)	0.1592 (4)	0.0242 (9)	
H4	0.6385	−0.1880	0.1506	0.029*	
N4	0.3485 (4)	−0.0610 (4)	0.1302 (4)	0.0256 (9)	
O1	0.6824 (5)	−0.6414 (4)	−0.3044 (4)	0.0562 (12)	
O2	0.5370 (4)	−0.5184 (3)	−0.3944 (3)	0.0269 (8)	
O3	0.2806 (4)	0.3234 (3)	0.7355 (3)	0.0326 (8)	
O4	0.2120 (4)	0.5109 (3)	0.6606 (3)	0.0333 (8)	
O1W	0.0693 (8)	−0.0278 (10)	0.0842 (10)	0.193 (5)	
O2W	0.9457 (7)	0.2012 (9)	0.7044 (9)	0.165 (4)	

### Atomic displacement parameters (Å^2^)

**Table:**

	*U*^11^	*U*^22^	*U*^33^	*U*^12^	*U*^13^	*U*^23^
Zn1	0.0281 (3)	0.0233 (3)	0.0200 (3)	−0.0011 (2)	−0.0043 (2)	−0.0080 (2)
C1	0.030 (3)	0.034 (3)	0.028 (3)	0.000 (2)	−0.011 (2)	−0.012 (2)
C2	0.023 (3)	0.038 (3)	0.038 (3)	0.001 (2)	−0.015 (2)	−0.011 (2)
C3	0.032 (3)	0.026 (3)	0.033 (3)	0.005 (2)	−0.013 (2)	−0.008 (2)
C4	0.027 (2)	0.017 (2)	0.019 (2)	−0.0010 (19)	−0.007 (2)	−0.0037 (19)
C5	0.029 (3)	0.022 (2)	0.019 (3)	0.002 (2)	−0.009 (2)	−0.008 (2)
C6	0.032 (3)	0.025 (3)	0.020 (3)	−0.006 (2)	−0.009 (2)	−0.003 (2)
C7	0.030 (3)	0.025 (2)	0.022 (3)	0.001 (2)	−0.011 (2)	−0.003 (2)
C8	0.032 (3)	0.031 (3)	0.040 (3)	0.002 (2)	−0.018 (3)	−0.009 (2)
C9	0.033 (3)	0.036 (3)	0.046 (4)	0.011 (2)	−0.019 (3)	−0.011 (3)
C10	0.028 (3)	0.030 (3)	0.037 (3)	0.009 (2)	−0.007 (2)	−0.010 (2)
C11	0.024 (2)	0.027 (3)	0.017 (3)	−0.004 (2)	−0.004 (2)	−0.001 (2)
C12	0.024 (2)	0.018 (2)	0.017 (2)	−0.0006 (19)	−0.003 (2)	−0.0024 (19)
C13	0.028 (3)	0.024 (3)	0.023 (3)	−0.001 (2)	−0.011 (2)	−0.001 (2)
C14	0.033 (3)	0.022 (2)	0.021 (3)	−0.004 (2)	−0.007 (2)	−0.003 (2)
C15	0.039 (3)	0.028 (3)	0.027 (3)	0.003 (2)	−0.019 (2)	−0.011 (2)
C16	0.034 (3)	0.025 (3)	0.033 (3)	0.003 (2)	−0.016 (2)	−0.005 (2)
C17	0.039 (3)	0.019 (2)	0.018 (3)	−0.004 (2)	−0.011 (2)	−0.0010 (19)
C18	0.033 (3)	0.025 (3)	0.021 (3)	−0.008 (2)	−0.012 (2)	0.001 (2)
C19	0.028 (3)	0.028 (3)	0.025 (3)	0.001 (2)	−0.008 (2)	−0.004 (2)
C20	0.036 (3)	0.024 (3)	0.029 (3)	0.000 (2)	−0.012 (2)	−0.004 (2)
C21	0.029 (3)	0.032 (3)	0.026 (3)	0.002 (2)	−0.003 (2)	0.001 (2)
C22	0.029 (3)	0.033 (3)	0.027 (3)	0.002 (2)	−0.002 (2)	−0.009 (2)
C23	0.025 (3)	0.038 (3)	0.022 (3)	0.002 (2)	−0.004 (2)	−0.010 (2)
C24	0.022 (2)	0.032 (3)	0.024 (3)	0.000 (2)	−0.008 (2)	−0.009 (2)
N1	0.026 (2)	0.022 (2)	0.022 (2)	−0.0031 (17)	−0.0075 (18)	−0.0031 (17)
N2	0.030 (2)	0.020 (2)	0.023 (2)	0.0034 (17)	−0.0070 (18)	−0.0079 (17)
N3	0.024 (2)	0.025 (2)	0.024 (2)	0.0051 (17)	−0.0101 (18)	−0.0102 (17)
N4	0.029 (2)	0.025 (2)	0.024 (2)	0.0014 (17)	−0.0113 (18)	−0.0052 (17)
O1	0.084 (3)	0.042 (2)	0.048 (3)	0.023 (2)	−0.038 (2)	−0.021 (2)
O2	0.0374 (19)	0.0286 (18)	0.0185 (18)	−0.0098 (15)	−0.0122 (16)	−0.0003 (14)
O3	0.0286 (18)	0.037 (2)	0.026 (2)	0.0072 (16)	−0.0025 (16)	−0.0083 (16)
O4	0.034 (2)	0.038 (2)	0.022 (2)	0.0040 (16)	−0.0042 (16)	−0.0012 (16)
O1W	0.133 (6)	0.237 (10)	0.239 (11)	0.082 (6)	−0.129 (7)	−0.141 (9)
O2W	0.077 (5)	0.214 (9)	0.183 (9)	0.004 (5)	−0.022 (5)	0.052 (7)

### Geometric parameters (Å, °)

**Table:**

Zn1—O2^i^	2.071 (3)	C13—N4	1.326 (6)
Zn1—O2^ii^	2.102 (3)	C13—N3	1.356 (6)
Zn1—N1	2.103 (4)	C13—C14	1.472 (6)
Zn1—N2	2.128 (4)	C14—C19	1.384 (7)
Zn1—O4	2.182 (3)	C14—C15	1.385 (6)
Zn1—O3	2.217 (4)	C15—C16	1.384 (7)
Zn1—C24	2.530 (5)	C15—H15	0.9300
C1—N1	1.322 (6)	C16—C17	1.390 (7)
C1—C2	1.396 (7)	C16—H16	0.9300
C1—H1	0.9300	C17—C18	1.396 (6)
C2—C3	1.371 (7)	C17—C20	1.499 (6)
C2—H2	0.9300	C18—C19	1.380 (6)
C3—C4	1.400 (6)	C18—H18	0.9300
C3—H3	0.9300	C19—H19	0.9300
C4—C12	1.415 (6)	C20—O1	1.216 (6)
C4—C5	1.424 (6)	C20—O2	1.310 (6)
C5—N3	1.370 (5)	C21—C22	1.388 (7)
C5—C6	1.379 (6)	C21—C23^iii^	1.392 (7)
C6—N4	1.374 (6)	C21—H23	0.9300
C6—C7	1.436 (6)	C22—C23	1.388 (7)
C7—C11	1.403 (6)	C22—H22	0.9300
C7—C8	1.410 (6)	C23—C21^iii^	1.392 (7)
C8—C9	1.366 (7)	C23—C24	1.494 (7)
C8—H8	0.9300	C24—O4	1.260 (6)
C9—C10	1.388 (7)	C24—O3	1.261 (5)
C9—H9	0.9300	N3—H4	0.8600
C10—N2	1.320 (6)	O2—Zn1^iv^	2.071 (3)
C10—H10	0.9300	O2—Zn1^ii^	2.102 (3)
C11—N2	1.358 (6)	O1—O2W^iv^	2.891 (9)
C11—C12	1.455 (6)	O1W—O2W^v^	2.886 (14)
C12—N1	1.352 (6)	O1W—N4	2.899 (10)
			
O2^i^—Zn1—O2^ii^	78.91 (13)	C4—C12—C11	120.6 (4)
O2^i^—Zn1—N1	100.31 (14)	N4—C13—N3	111.6 (4)
O2^ii^—Zn1—N1	101.54 (14)	N4—C13—C14	127.0 (4)
O2^i^—Zn1—N2	171.64 (14)	N3—C13—C14	121.4 (4)
O2^ii^—Zn1—N2	93.17 (14)	C19—C14—C15	118.3 (4)
N1—Zn1—N2	78.50 (14)	C19—C14—C13	121.7 (4)
O2^i^—Zn1—O4	90.24 (13)	C15—C14—C13	120.0 (4)
O2^ii^—Zn1—O4	96.74 (13)	C16—C15—C14	121.4 (5)
N1—Zn1—O4	160.32 (14)	C16—C15—H15	119.3
N2—Zn1—O4	93.37 (14)	C14—C15—H15	119.3
O2^i^—Zn1—O3	92.56 (13)	C15—C16—C17	120.2 (5)
O2^ii^—Zn1—O3	155.21 (12)	C15—C16—H16	119.9
N1—Zn1—O3	102.89 (14)	C17—C16—H16	119.9
N2—Zn1—O3	95.77 (14)	C16—C17—C18	118.4 (4)
O4—Zn1—O3	59.73 (13)	C16—C17—C20	119.3 (4)
O2^i^—Zn1—C24	90.48 (14)	C18—C17—C20	122.3 (4)
O2^ii^—Zn1—C24	126.02 (15)	C19—C18—C17	120.6 (4)
N1—Zn1—C24	132.44 (16)	C19—C18—H18	119.7
N2—Zn1—C24	96.40 (15)	C17—C18—H18	119.7
O4—Zn1—C24	29.87 (14)	C18—C19—C14	121.0 (4)
O3—Zn1—C24	29.89 (14)	C18—C19—H19	119.5
N1—C1—C2	122.6 (4)	C14—C19—H19	119.5
N1—C1—H1	118.7	O1—C20—O2	124.2 (5)
C2—C1—H1	118.7	O1—C20—C17	120.2 (5)
C3—C2—C1	119.6 (4)	O2—C20—C17	115.6 (4)
C3—C2—H2	120.2	C22—C21—C23^iii^	120.2 (5)
C1—C2—H2	120.2	C22—C21—H23	119.9
C2—C3—C4	118.9 (4)	C23^iii^—C21—H23	119.9
C2—C3—H3	120.5	C21—C22—C23	120.3 (5)
C4—C3—H3	120.5	C21—C22—H22	119.9
C3—C4—C12	118.2 (4)	C23—C22—H22	119.9
C3—C4—C5	126.3 (4)	C22—C23—C21^iii^	119.5 (5)
C12—C4—C5	115.5 (4)	C22—C23—C24	120.4 (4)
N3—C5—C6	105.5 (4)	C21^iii^—C23—C24	120.0 (5)
N3—C5—C4	129.5 (4)	O4—C24—O3	120.6 (4)
C6—C5—C4	124.8 (4)	O4—C24—C23	120.1 (4)
N4—C6—C5	110.1 (4)	O3—C24—C23	119.2 (5)
N4—C6—C7	129.9 (4)	O4—C24—Zn1	59.6 (2)
C5—C6—C7	120.0 (4)	O3—C24—Zn1	61.2 (2)
C11—C7—C8	117.4 (4)	C23—C24—Zn1	174.0 (3)
C11—C7—C6	117.2 (4)	C1—N1—C12	119.0 (4)
C8—C7—C6	125.4 (4)	C1—N1—Zn1	127.1 (3)
C9—C8—C7	119.3 (5)	C12—N1—Zn1	113.7 (3)
C9—C8—H8	120.4	C10—N2—C11	118.4 (4)
C7—C8—H8	120.4	C10—N2—Zn1	127.5 (3)
C8—C9—C10	119.4 (5)	C11—N2—Zn1	113.5 (3)
C8—C9—H9	120.3	C13—N3—C5	107.5 (4)
C10—C9—H9	120.3	C13—N3—H4	126.2
N2—C10—C9	123.1 (5)	C5—N3—H4	126.2
N2—C10—H10	118.5	C13—N4—C6	105.3 (4)
C9—C10—H10	118.5	C20—O2—Zn1^iv^	132.2 (3)
N2—C11—C7	122.4 (4)	C20—O2—Zn1^ii^	125.3 (3)
N2—C11—C12	115.9 (4)	Zn1^iv^—O2—Zn1^ii^	101.09 (13)
C7—C11—C12	121.8 (4)	C24—O3—Zn1	88.9 (3)
N1—C12—C4	121.7 (4)	C24—O4—Zn1	90.6 (3)
N1—C12—C11	117.7 (4)		

Symmetry codes: (i) *x*, *y*+1, *z*+1; (ii) −*x*+1, −*y*, −*z*; (iii) −*x*, −*y*+1, −*z*+2; (iv) *x*, *y*−1, *z*−1; (v) −*x*+1, −*y*, −*z*+1.

### Hydrogen-bond geometry (Å, °)

**Table:**

*D*—H···*A*	*D*—H	H···*A*	*D*···*A*	*D*—H···*A*
N3—H4···O3^v^	0.86	2.10	2.781 (5)	136.

Symmetry codes: (v) −*x*+1, −*y*, −*z*+1.
